# Effects of treatment dosage of whole‐body cryotherapy upon post‐match recovery of endocrine and biochemical markers in elite rugby league players: An experimental study

**DOI:** 10.1002/hsr2.1227

**Published:** 2023-04-19

**Authors:** Adam S. Naylor, Ben J. Edwards, Colin M. Robertson

**Affiliations:** ^1^ Sports Injuries Research Group Edge Hill University Ormskirk UK; ^2^ Liverpool John Moores University Liverpool UK; ^3^ UTS Foundation Hoylake Wirral UK

**Keywords:** recovery, whole‐body cryotherapy, rugby league, fatigue marker

## Abstract

**Background and Aims:**

The use of whole‐body cryotherapy (WBC) for athletic recovery is becoming increasingly popular despite the lack of evidence supporting the dosage parameters in its implementation. The aim of the current study was to investigate the dose–response effects of WBC following match‐play in elite rugby league players.

**Methods:**

We observed endocrine (salivary cortisol and testosterone) and biochemical (creatine kinase) responses following three separate post‐match recovery periods in elite rugby league players. Comparisons were made between a single exposure (3 min at −120°C to ^‐^−135°C) of WBC to two consecutive exposures (2 × 3 min), to a control (no exposure) during the recovery trials. Recovery characteristics were measured 36 h prematch, immediately postmatch, and 60 h postmatch.

**Results:**

Cortisol concentrations remained unchanged in its pattern of response during the postmatch recovery periods across all WBC doses. Testosterone concentrations increased significantly (*p* < 0.0005) at 60 h, in the WBC2 trial. The Testosterone:Cortisol ratio increased significantly (*p* < 0.0005) at 60 h in the WBC2 trial, while during the WBC0 trial it did not recover to baseline levels. No significant effect on creatine kinase concentration was observed, although a statistical trend was shown in WBC2 for improved reduction of this marker at 60 h.

**Conclusions:**

These findings suggest that two, consecutive exposures to WBC immediately following fatiguing rugby league competition appear to stimulate an increase to the anabolic endocrine profile of participants by 60 h post‐match, and may reduce the CK concentration. Coaches and athletes should consider the treatment dosage of WBC when used to optimize the desired response following a high‐stress environment.

## 1 INTRODUCTION

Optimizing recovery within a contact sports environment such as rugby league (RL) is of high importance in preparing athletes for competition as well as maintaining health and well‐being. Regular fixtures and high‐density training schedules induce high levels of fatigue, and therefore competing while excessively fatigued can heighten the risk of injury.^1^, ^2^ Time‐loss injuries create a heavy financial burden for RL teams in addition to the physical and mental impact of being unavailable for competition and training.^3^

RL is an intermittent, high‐intensity team collision sport consisting of 13 players per team played over an 80‐min match of two 40‐min halves.^4^ Players cover distances of between 8 and 10 km during a match, with up to 10% of this consisting of repeated (short distance) high‐speed running, accelerating and decelerating, added to repeated tackling events during the game.^5^ Collectively, these activities cause a distinct alteration of homeostatic body status which may take between 48 h and 120 h to restore to a precompetition level.^6^, ^7^ This disruption is suitably reflected by biochemical and endocrinological profiles, which indicate cellular disruption and hormonal disturbance.^8^ In applied studies, creatine kinase (CK) is frequently used as an indirect marker of muscle damage to represent the former, with cortisol (C) and testosterone (T) concentration markers employed to represent the latter.^9^, ^10^

Of the many recovery modalities seen as common practice in RL (such as active recovery, cold water immersion, contrast water immersion, or compression garments), whole‐body cryotherapy (WBC) is a largely under‐researched, yet popular inclusion to rugby athletes’ schedules.^8^, ^11^ WBC is a short duration (2–3 min) exposure to extreme cold air of −110°C to −180°C in temperature‐controlled units, either through electric cooling systems or by means of liquid nitrogen.^12^, ^13^ Previous studies involving sport and non‐sport populations have observed reduced symptoms of pain and stiffness as well as improved ratings of sleep quality, general feelings of well‐being, and recovery from exercise.^14^, ^15^, ^16^, ^17^

WBC is typically applied post‐activity as a singular treatment exposure, or in a series of exposures over several days during a recovery period.^18^, ^19^ With the intention to create a greater immediate thermal stress, two studies have used *consecutive* exposures of WBC within a single treatment session following physically fatiguing activity, that is, with a short, thermo‐neutral re‐warming period between each exposure. These studies have shown inconclusive benefits to recovery.^20^, ^21^ The temperature exposure (2 × 3 min at −85°C with 5 min re‐warming between exposures) employed in both studies was higher than typically applied in other WBC studies (<110°C). As such, it is not clear whether (i) short‐term, consecutive exposures of WBC provide additional benefit to recovery, and (ii) repeating the exposure to WBC at lower extreme temperatures (<110°C) is any more beneficial than warmer (i.e., −85°C) temperatures.

As such, this is the first study to investigate the effects of WBC (<110°C) over a post‐match recovery period on the profiling of hormonal and biochemical markers in RL players. The aim of this study was to compare these responses when the immediate (<1 h) postmatch routines contained either no WBC exposure, a single exposure to WBC, or two *consecutive* exposures to WBC. In light of the current evidence, a null hypothesis stating that no influence of WBC exposure upon the recovery process was tested.

## 2 MATERIALS AND METHODS

### 2.1 Participants

Twenty‐three elite RL players (mean ± SD: age 28.0 ± 4.9 years; height 1.84 ± 0.10 m; weight 96.0 ± 9.6 kg) from a European Super League (ESL) team volunteered to participate in the study. Before the study all participants were briefed on the purpose, procedures, and benefits of the study. Informed consent was obtained from all participants, with institutional ethical approval from the host Departmental Research Ethics Committee and in accordance with the Helsinki Declaration.

### 2.2 Experimental approach

**Figure 1:**
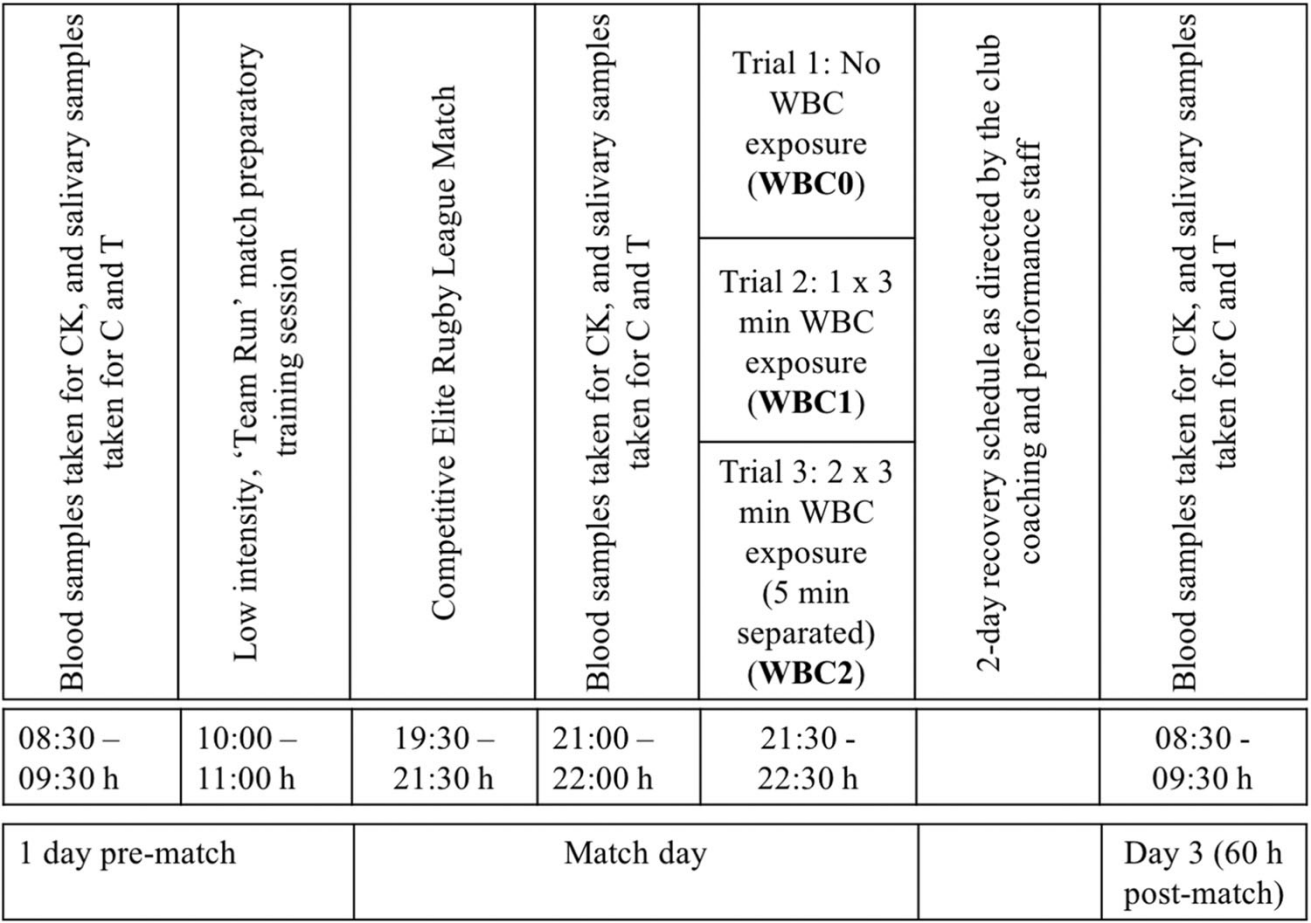
Study schematic showing sampling times and experimental trials over a 5‐day period.

The participants were monitored over three competitive evening matches and their subsequent recovery periods to observe biochemical (CK) and hormonal (salivary testosterone and cortisol) responses following varied exposure to WBC. Match cycles followed the sample time structure outlined in the schematic in Figure 1. The three post‐match intervention protocols were referred to as WBC0, WBC1, and WBC2, reflecting the number of WBC exposures involved in the post‐match period. The WBC1 and WBC2 trials were integrated into the team's typical recovery schedule as dictated by the club's performance staff, and, therefore, the experimental variable (i.e., WBC) was not randomized in terms of its application to participants. The sample time points used in each individual recovery cycle were used to generate a temporal response for that experimental trial over a 60‐h period, including 36 h pre‐match (baseline), and immediate (<20 min) postmatch measures. For comparative analysis between cycles, a repeated measures approach was adopted, whereby players who were involved in all three cycles were used for analysis. As a result of changes in team selection, injury, and athlete scheduling across the pre‐match, postmatch, and 60‐h time point measures, participant numbers were finalized at *n* = 11 and *n* = 6 for salivary hormone and CK analyses, respectively, across the three trials. All sampling occurred during April and June during a regular ESL season.

### 2.3 WBC procedures

A mobile trailer‐mounted liquid nitrogen cryochamber (Sappari), owned and operated by British Oxygen Company Linde (BOC), was used for all WBC treatments. All participants consented to the use of the cryochamber according to the guidelines set by BOC and were cleared of any contraindications according to the BOC mandate by the club doctor. Participants donned the appropriate safety attire consisting of shorts, thick socks, sandals, hat, gloves, elbow and knee joint elasticated bandage coverings, and face mask covering the nose and mouth. Within 1 h after match completion, and after post‐match sampling, participants entered the cryochamber via an initial acclimatization chamber cooled to −60°C for 30 s. They then entered the main chamber cooled to between −120°C and −135°C for 3 min. These application parameters are recommended to create sufficient thermal gradients following the removal of the WBC stimulus.^22^

For the treatment protocol involving two WBC treatments, the participants had 5 min between WBC treatments standing in atmospheric temperatures (15°C to 20°C) outside of the cryotherapy chamber before re‐entering.

### 2.4 Saliva sampling and analysis procedures

The participants were asked to refrain from eating or drinking in the hour before sample collection to ensure regular salivary content and quality. Compliance was checked when participants arrived at the training venue via questioning the timing of their last meal or drink. Compliance was shown to be high by the fact that all samples were able to be collected within a 1‐h period upon player arrival, and before starting training sessions. On match days, participants continued their typical match preparations in terms of nutrition, mainly comprising of sports recovery/energy drinks ingested during the game so as not to impact performance in any way.

For post‐match samples, no drinks other than water were ingested until sampling was complete. For all samples taken, water (100 mL, 15°C to 20°C) was provided 5–10 min before sample collection to rinse the mouth. It was only upon rigorous adherence to these steps that a sample was considered suitable, and reliable for the purpose of analysis. A salivette oral swab (Salimetrics LLC) was used to collect unstimulated saliva, placed beneath the tongue for 3–4 min. Salivettes were then placed in individual storage tubes (Salimetrics LLC), immediately placed on ice and frozen within 1 h at <^‐^20°C until required for analysis. Samples were labeled with individual coding so that participant data was pseudonymized and allowed for a single‐blind sample analysis process.

For analysis preparation, samples were defrosted and centrifuged at 5600 rpm for 5 min. All saliva samples deemed reliable upon collection, and also deemed usable following sample analysis, were included in the statistical data presented. C (μg/dL) and T (pg/mL) were assayed and analyzed in duplicate using commercially available enzyme‐linked immunosorbent assay (ELISA) kits (Salimetrics LLC). Microplate analysis was carried out using a microplate reader with Gen‐5 software (ELx‐800, Biotek Instruments Inc.). Standard curves were constructed as per the manufacturer's instructions, standards, and sample controls (Salimetrics, LLC).

T/C ratio was determined by dividing the concentration of T by the concentration of C. The manufacturer‐determined minimum detection limit for T was 1.0 pg/mL (relative 0.0001 μg/dL), with an average intra‐ and inter‐assay coefficient of variance (CV) of 4.1 ± 4.7% and <12%, respectively. The manufacturer‐determined minimum detection limit for C was 0.007 μg/dL (relative 70 pg/mL), with average intra‐ and inter‐assay CV of 4.3 ± 4.0% and <12%, respectively. CV was calculated per duplicate sample during microplate analysis and provided by the Gen‐5 data analysis output.

### 2.5 Creatine kinase sampling and analysis procedures

CK was measured via 30 μL capillaries whole‐blood fingertip samples using spring‐loaded, disposable single‐use lancets. Samples were applied to pre‐calibrated Reflotron CK analysis test strips and analyzed immediately using a Reflotron Plus spectrophotometer (Boehringer Mannheim). Reflotron Precinorm U Quality Control sample tests were administered before all sample collection periods. Participant samples were collected as per the schematic in Figure 1.

### 2.6 Global position system (GPS) data

GPS measures were taken using portable GPS devices (SPI‐Pro; 5 Hz, GPSports) and an inbuilt triaccelerometer (100 Hz) to compare the relative match intensity across the three fixtures. Players were accustomed to wearing GPS devices during training and matches. Each player was pre‐fitted with an appropriately sized vest housing the portable GPS unit between the scapulae underneath their team shirt. Players wore the vest and unit for the warm‐up and match period. All data were downloaded to a computer using SPI Ezy and analyzed using Team AMS software (GPSports). Total distance covered (m) and total impacts over 8Gs of force were extracted for analysis.

### 2.7 Statistical analysis

Data were analyzed using the Statistical Package for Social Sciences (SPSS) for Windows (SPSS), IBM version 26. Differences in the raw data between the extent of WBC exposure following each match were evaluated using a general linear model (GLM) with repeated measures, within the subject factor “exposure to WBC” (three levels) and within subject factor ‘time point of measure (three levels); and interaction. To correct violations of sphericity, the degrees of freedom were corrected using Huynh–Feldt (*ε* > 0.75) or Greenhouse‐Geisser (*ε* < 0.75) values for *ε*, as appropriate. Effect sizes (ES) were calculated from the ratio of the mean difference to the pooled standard deviation. The magnitude of ES was classified as trivial (≤0.20), small (>0.20–0.60), moderate (>0.60–1.20), large (>1.20–2.00), and very large (>2.00) based on the guidelines from Batterham and Hopkins.^23^ A one‐way analysis of variance (ANOVA) for independent groups was used to verify that the relative intensity over the three matches was similar, since team selection was not fully consistent for each match. All data remained in original scale measures for statistical analyses and presented as mean ± SD unless otherwise stated. A relative percentage change from baseline (36‐h pre‐match) measures was also calculated.

A calculation for the critical difference was employed to discriminate individual variation from those associated with the application of WBC, using the formula:

$$C{D_{95}}^{\%}=2.77\times \surd \left(C{V_{a}}^{2}+C{V_{i}}^{2}\right)$$

where CV_a_ = analytical coefficient of variation and CV_i_ = CV of within‐subject biological variation.

**Figure 2:**
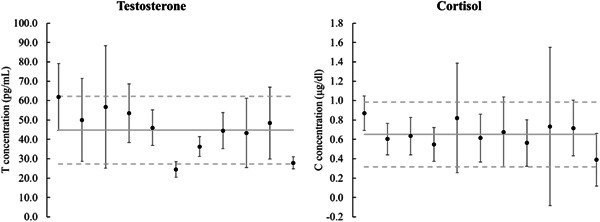
Individual variability of salivary hormone responses at 36‐h pre‐match across the three trials. Solid line represents the mean within‐participant biological coefficient of variation with dashed lines at ±1SD.

CV_a_ utilized the intra‐assay CV values for T (4.1%) and C (4.3%). CV_i_ was calculated as the average within‐participant CV of baseline measures (i.e., 36 h pre‐match) for T (28.8%) and C (48.8%) across the three experimental trials (Figure 2). The critical difference at 95% confidence limit for T was calculated at 85.5%, and 135.6% for C. These data implied that in order for significant effects to be considered, the change in T or C concentrations were to be in excess of the percentage change in the measured hormone.

A post hoc power analysis confirmed that >10 participants were sufficient for a power of 0.8, *α* level of 0.05, a moderate effect size of *d* = 0.6 with critical *F* = 3.64. Regrettably, the CK analysis was unable to reach this and presents as a low‐powered analysis of 1−*β* = 0.4.

Statistical significance was set at an alpha level of 5% (*p* < 0.05) for two‐tailed analyses. When the SPSS output demonstrated significant levels of *p* = 0.000, these were corrected to *p* < 0.0005.^24^

## 3 RESULTS

A summary of match GPS data is provided in Table 1, all salivary and biochemical marker concentrations in Table 2, and between‐trial effect size (Cohen's *d*) comparisons at the 60‐h time point in Table 3.

**Table 1:** Mean ± SD values for GPS data from each match fixture.ta.

Match cycle	Total distance (m) covered per player	Number of impacts over 8G per player
WBC0	7721 ± 1005	105 ± 57
WBC1	8291 ± 1269	87 ± 41
WBC2	7540 ± 1427	101 ± 54

**Table 2:** Mean ± SD values for hormonal and biochemical markers at all measured time points and conditions.

	36 h pre‐match	20 min post‐match	60 h post‐match
**T (pg/mL) (*n* = 11)**			
WBC0	50.7 ± 24.9	54.3 ± 19.1	37.5 ± 11.8
WBC1	39.7 ± 11.9	42.4 ± 13.7	54.0 ± 17.4
WBC2	44.1 ± 11.9	49.2 ± 25.3	107.2 ± 27.3**
**C (μg/dL) (*n* = 11)**			
WBC0	0.51 ± 0.29	1.08 ± 0.66*	0.43 ± 0.24
WBC1	0.93 ± 0.37	1.44 ± 0.56*	0.63 ± 0.26
WBC2	0.52 ± 0.23	0.95 ± 0.57*	0.68 ± 0.39
**T/C ratio (*n* = 11)**			
WBC0	99.4 ± 28.0	61.7 ± 31.2	92.5 ± 37.3
WBC1	47.6 ± 19.3	31.2 ± 10.4	87.8 ± 25.5
WBC2	95.6 ± 30.1	62.4 ± 25.4	179.4 ± 54.1**
**CK (U/L) (*n* = 6)**			
WBC0	328 ± 162	506 ± 204	481 ± 196
WBC1	333 ± 89	425 ± 75	507 ± 180
WBC2	461 ± 133	555 ± 132	382 ± 146

* Significantly different (*p* < 0.05) to 36 h pre‐match and 60 h post‐match.

** Significant interaction effect (*p* < 0.0005).

**Table 3:** Effect size (Cohen's *d*) of observed differences between trials at 60 h post‐match.

Comparison	T	T/C	CK
60 h WBC2 vs. WBC1	2.32	2.01	0.76
60 h WBC2 vs. WBC0	3.31	1.66	0.57
60 h WBC1 vs. WBC0	1.11	0.23	0.14

*Note*: Effect sizes (ES) represent: trivial (≤0.20), small (>0.20–0.60), moderate (>0.60–1.20), large (>1.20–2.00), and very large (>2.00). ES was calculated using original values.

### 3.1 GPS variability

Global positioning system match data for 19 different players across the three trials were included. Due to a failure of the GPS recording unit during the matches, data from four players were not included in this analysis. The one‐way ANOVA for independent groups revealed no statistically significant difference across all three matches for both, total distance covered or total number of impacts over 8Gs of force (*F*_2,29_ = 0.99, *p* = 0.38 and *F*_2,29_ = 0.33, *p* = 0.73 respectively). A Levene's test showed homogeneity of variance across the three matches for both measures (*p* = 0.49, *p* = 0.44, respectively). Overall, it was assumed that a similar physiological demand was observed across the three matches (Table 1).

### 3.2 Testosterone responses

For the analysis of T responses, the GLM revealed a significant effect for condition (*F*_2,20_ = 13.1, *p* < 0.0005). Pairwise comparisons showed that the WBC2 condition was significantly higher than WBC0 (mean difference = 19.3 pg/mL, 95% CI = 5.6–33.0, *p* = 0.007; ES = 0.63) and WBC1 (21.4 pg/mL, 95% CI = 10.6–32.3, *p* = 0.001; ES = 0.72). There was no statistical difference between the WBC0 and WBC1 conditions (*p* = 1.000).

**Figure 3:**
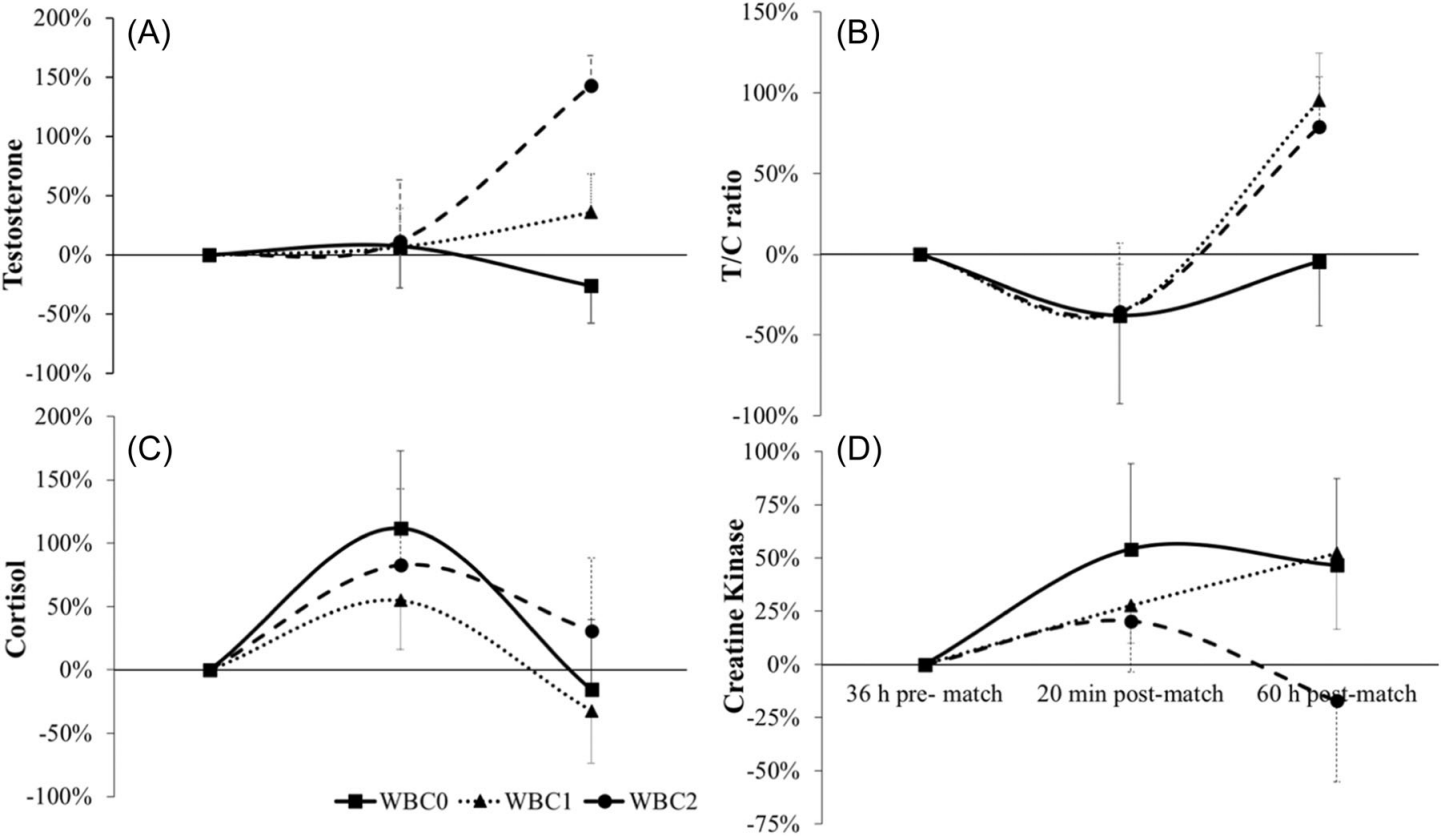
Percentage changes of recovery markers from 36 h pre‐match baseline for all trials (A ‐ testosterone; B ‐ T/C ratio; C ‐ cortisol; D ‐ CK).

A significant effect for time was also evident (*F*_1.9,18.9_ = 15.4, *p* < 0.0005). Pairwise comparisons showed that the overall measure of T concentration at 60‐h post‐match was significantly higher than that at baseline (36 h pre‐match, mean difference = 21.4 pg/mL, 95% CI = 12.7–30.1, *p* < 0.0005; ES = 0.61) and immediately post‐match (mean difference = 17.6 pg/mL, 95% CI = 3.8–31.4, *p* = 0.013; ES = 0.53). There was no statistical difference between the baseline and post‐match time point measures for T (*p* = 1.000). A significant interaction was found at the 60‐h time point (*F*_2.2,22.1_ = 21.9, *p* < 0.0005, see Figure 3A). From baseline values, increases of 143% during WBC2 (107.2 pg/mL, 95% CI = 88.9–125.5 pg/mL) and 36% during WBC1 (54.0 pg/mL, 95% CI = 42.4–65.7 pg/mL) were observed. A *decrease* by 26% (37.5 pg/mL, 95% CI = 29.6–45.4 pg/mL) was evident during WBC0 at the 60‐h time point.

### 3.3 Cortisol responses

For the analysis of C responses, the GLM revealed a significant effect for condition (*F*_1.3,13.1_ = 4.6, *p* = 0.043). Pairwise comparisons showed that the WBC1 condition was significantly higher than WBC2 (mean difference = 0.31 μg/dL, 95% CI = 0.12–0.49, *p* = 0.002: ES = 0.64). There was no statistical difference between the WBC0 and WBC1 (*p* = 0.182) and WBC0 and WBC2 conditions (*p* = 1.000).

A significant effect for time was also evident (*F*_1.2,11.9_ = 21.2, *p* < 0.0005). Pairwise comparisons showed that the overall measure of C concentration immediately post‐match was significantly higher than that at baseline (36‐h pre‐match, mean difference = 0.51 μg/dL, 95% CI = 0.20–0.81, *p* = 0.002; ES = 0.45) and at 60‐h (mean difference = 0.56 μg/dL, 95% CI = 0.22–0.89, *p*= 0.002; ES = 0.52). There was no statistical difference between the baseline and 60‐h post‐match time point measures for C (*p* = 1.000). There was no significant interaction effect shown (*p* = 0.091, see Figure 3C).

### 3.4 Testosterone/Cortisol (T/C) ratio responses

For the analysis of T/C ratio responses (Figure 3B), the GLM revealed a significant effect for condition (*F*_1.9,18.5_ = 24.7, *p* < 0.0005). Pairwise comparisons showed that the WBC2 condition was significantly higher than WBC0 (mean difference = 27.9, 95% CI = 0.6–55.3, *p* = 0.045; ES = 0.53) and WBC1 (56.9, 95% CI = 32.5–81.4, *p* < 0.0005; ES = 1.01). WBC0 was significantly higher than WBC1 (mean difference = 29.0, 95% CI = 12.2–45.8, *p*= 0.002: ES = 0.81). A significant effect for time was also evident (*F*_1.4,13.7_ = 59.9, *p* < 0.0005). Pairwise comparisons showed that the overall measure of the T/C ratio immediately post‐match significantly decreased compared to baseline values (36 h pre‐match, mean difference = 29.1, 95% CI = 17.7–40.4, *p* < 0.0005; ES = 0.21). The relationship value at 60‐h post‐match was significantly higher than that at baseline (mean difference = 39.1, 95% CI = 20.9–57.3, *p* < 0.0005; ES=0.45) and immediately post‐match (mean difference = 68.1, 95% CI = 45.7–90.6, *p* < 0.0005; ES = 0.54).

A significant interaction was found at the 60‐h time point (*F*_2.7,26.9_ = 9.0, *p* < 0.0005, see Figure 3B). From baseline values, this equates to an increase of 88% during WBC2 (179.4, 95% CI = 143.1–215.8), 85% during WBC1 baseline (87.5, 95% CI = 70.7–104.9), and a *decrease* by 7% (92.5, 95% CI = 80.5–118.2).

### 3.5 Creatine kinase

For the analysis of creatine kinase (CK) responses, the GLM revealed a lack of significance for condition (*F*_1.5,7.5_ = 1.18, *p* = 0.340) and time (*F*_1.4,7.2_ = 3.2, *p* = 0.109). The interaction effect showed a statistical trend (*F*_2.0,10.0_ = 3.24, *p* = 0.082). Figure 3D illustrates the CK responses across the three trials. The potential for an interaction effect is more evident when a difference from baseline values is expressed. At 60 h, the CK concentration during WBC2 was 17% *below* the baseline concentration 36 h pre‐match. In comparison, the percentage differences during WBC0 and WBC1 at 60 h were 47% and 52% *above* baselines, respectively.

## 4 DISCUSSION

The main finding of this study was that exposure to WBC within 1 h of match completion irrespective of one or two doses (WBC1 and WBC2), resulted in an 85%–90% elevation relative to baseline in T/C ratio by 60 h post‐match. This is potentially representative of an anabolic hormonal profile, and did not appear in the WBC0 trial. Regeneration of damaged tissue is driven by an anabolic environment, and so, therefore, the earlier that this is initiated during the recovery period, the greater the potential for more productive training sessions and future match preparation. However, it should be noted that whilst the T/C ratio is useful in observing the relative balance of each hormone, this value is merely a surrogate that is associated with or without fatigue states. As such, the T/C ratio should be interpreted in view of the behavior of each of its components. It has previously been shown that changes from more relative catabolic states to anabolic states during a post‐match recovery period (without WBC influence) are driven through greater changes in C rather than T.^6^, ^25^ Hayes et al.^26^ showed that normal daily hormonal rhythms also display this pattern. However, in the present study, moderate to very large effect sizes (ranging from *d* = 1.11 to *d* = 3.31) in T response was observed during the WBC2 trial in comparison the WBC0 and WBC1 trials, whilst C responses were similar across all three trials (Figure 3C). Only Russell et al.^27^ has previously shown increases in T after high intensity activity in academy level footballers, however, this data is only evident up to 24 h post‐repeated sprint activity. Grasso et al.^28^ noted a significant increase in T concentration following 14 sessions of WBC over a seven‐day period during a rugby union international team training camp. Unfortunately, the data from Grasso et al.^28^ is limited to analyses taken at two‐time points (day one and seven) following two daily sessions of WBC. Nevertheless, the significant increase in T concentration following exposure to WBC in all three studies (inclusive of the present study) shows a promising stimulus of endocrinological function. The mechanism by which this has occurred, unfortunately still cannot be explained by the current data, but the assumption that stimulation of the hypothalamic‐pituitary‐gonadal (HPG) axis has occurred certainly warrants further investigation.

Relative to baseline values, and as expected, a catabolic state immediately post‐match was observed in all three trials. During the WBC0 trial, an anabolic hormonal profile was not reached at 60 h postmatch (Figure 3B). Similarly, the data from McLellan et al.^6^ showed a T/C relationship that barely reached baseline levels up to 120 h postmatch, implying that a catabolic state was being maintained during this time. A persistent catabolic state should be considered detrimental for RL players, especially during short time periods between matches.

The C concentrations throughout each experimental cycle showed similar responses following rugby league competition. Therefore, it can be assumed that the HPA stimulation as a result of the intensity of each match was similar despite a lack of counterbalancing of trials. This acute C elevation is an important response in responding to high‐level psychobiological demands.^7^, ^29^ A similar pattern of C normalization was also observed by McLellan et al.^10^ where a return to baseline levels was achieved between 24 and 48 h. The consistency of C concentration responses in all three trials in this study may suggest that WBC has no short‐term effect upon the acute stress response via the HPA axis following high‐intensity RL competition.

Whilst unable to establish a statistically significant analysis, there are indications in Figure 3D that WBC2 provided the greatest influence upon CK appearance in the blood, but reflecting small to moderate effect sizes (*d* = 0.57–0.76). However, it would be inaccurate to assume that WBC concurrently influences a blood marker of muscle damage with the same sensitivity or mechanism as salivary hormone concentration since the characteristics of appearance will vary across the medium used. Furthermore, given that hormonal concentrations are centrally mediated via hypothalamic axes, and CK concentration is an indirect peripheral cell damage marker, it would be prudent to assess the effects of WBC upon salivary and blood markers separately and not directly in association. Furthermore, given the large variability of the number of impacts across player position (range 38–192 impacts) during a game, some matches could be deemed physically less intense for some individuals than others. The unpredictability of the live situation makes the direct comparison across matches challenging even on an individual level.

### 4.1 Limitations

It should be noted that in aiming to provide a “real world” applied context in RL, the participants acted as their own controls, and therefore to maintain a repeated measures approach, participant numbers were difficult to maximize due to team selection, injury, and absence. As such, the comparisons across and within trials became limited to 6–11 participants. In addition to this, it was assumed that the team followed an otherwise typical recovery schedule following the matches featured across the three different sampling trial periods. This included a variety of active recovery exercise, soft tissue massage and flexibility work. Every effort was made by the team staff to replicate the recovery routine where possible, whilst being dictated by the busy season schedule.

Consideration should also be given to the scheduling of sample collection. The circadian rhythm of C and T is such that there is a potential for large variation between morning and evening samples.^29^ C and T concentrations peak early in the morning (06:00 h), and are at their lowest at 23:00 h, with C demonstrating larger variation (up to 92%) compared to T (42%). In this study, samples were taken between 08:30 and 09:30 h during all sample days, except match day, where samples were taken at approximately 20:00 to 22:00 h. As a result, this may cause some difficulty in analyzing the complete effect of WBC, as we are also only providing daily ‘snapshots’ of endocrine variation that may not fully represent the overriding response.^30^ Future studies should aim to establish a more periodic reflection of RL player endocrine patterns (e.g. morning and evening measures) so that match play responses can be directly compared as opposed to estimated, especially when modalities such as WBC are applied. In this study, broad between‐participant variability was expressed via large standard deviations, furthering the need for knowledge of ‘true’ individual baselines.

Finally, this study specifically observed biochemical and hormonal markers of fatigue, and did not collect measurements representing other contributing mechanisms to the fatigue experience and recovery process. Future studies are encouraged to incorporate subjective or perceived ratings of fatigue (e.g., via questionnaire or visual analogue scaled ratings), indicators of neuromuscular function and inflammatory responses to better contextualize the internal physiological responses to experiential data.

## 5 PERSPECTIVE

This study has observed statistically significant and large to very large effect size differences in T at 60 h following two exposures of WBC for three minutes, administered immediately after competitive RL match play. This increase was also the main influence supporting a high relative anabolic hormonal balance within 60 h of a competitive match (in comparison to pre‐match baselines), which may benefit tissue regeneration and optimize recovery between match cycles. Less conclusive effects of a single dose of WBC immediately following a match were observed and so practitioners, coaches and athletes should consider treatment dosage to optimize the desired response following a high‐stress environment.

## AUTHOR CONTRIBUTIONS

**Adam S. Naylor**: Conceptualization; data curation; formal analysis; funding acquisition; investigation; methodology; project administration; resources; software; writing—original draft; writing—review & editing. **Ben J. Edwards**: Writing—review & editing. **Colin M**. **Robertson**: Conceptualization; funding acquisition; resources; supervision; writing—review & editing.

## CONFLICT OF INTEREST STATEMENT

The authors declare no conflict of interest.

## TRANSPARENCY STATEMENT

The lead author Adam S. Naylor affirms that this manuscript is an honest, accurate, and transparent account of the study being reported; that no important aspects of the study have been omitted; and that any discrepancies from the study as planned (and, if relevant, registered) have been explained.

## Data Availability

The data that support the findings of this study are available from the corresponding author upon reasonable request.
